# Effects of multi-strain probiotic supplementation on intestinal microbiota, tight junctions, and inflammation in young broiler chickens challenged with *Salmonella enterica* subsp. *enterica*

**DOI:** 10.5713/ajas.19.0427

**Published:** 2019-11-12

**Authors:** Chi Huan Chang, Po Yun Teng, Tzu Tai Lee, Bi Yu

**Affiliations:** 1 Department of Animal Science, National Chung Hsing University, Taichung City 402, Taiwan; 2 The iEGG and Animal Biotechnology Center, National Chung Hsing University, Taichung, 402, Taiwan

**Keywords:** Multi-strain Probiotics, Intestinal Microbiota, Tight Junctions, Inflammation, *Salmonella enterica* subsp. *Enterica*

## Abstract

**Objective:**

This study assessed the effects of probiotics on cecal microbiota, gene expression of intestinal tight junction proteins, and immune response in the cecal tonsil of broiler chickens challenged with *Salmonella enterica* subsp. *enterica*.

**Methods:**

One-day-old broiler chickens (n = 240) were randomly allocated to four treatments: negative control (Cont), multi-strain probiotic-treated group (Pro), *Salmonella*-infected group (Sal), and multi-strain probiotic-treated and *Salmonella*-infected group (ProSal). All chickens except those in the Cont and Pro groups were gavaged with 1×10^8^ cfu/mL of *S. enterica* subsp. *enterica* 4 days after hatching.

**Results:**

Our results indicated that body weight, weight gain, and feed conversion ratio of birds were significantly reduced (p<0.05) by *Salmonella* challenge. Chickens challenged with *Salmonella* decreased cecal microbial diversity. Chickens in the Sal group exhibited abundant *Proteobacteria* than those in the Cont, Pro, and ProSal groups. *Salmonella* infection downregulated gene expression of *Occludin*, zonula occludens-1 (*ZO1*), and *Mucin 2* in the jejunum and *Occludin* and *Claudin* in the ileum. Moreover, the Sal group increased gene expression of interferon-γ (*IFN-γ*), interleukin-6 (*IL-6*), *IL-1β*, and lipopolysaccharide-induced tumor necrosis factor-alpha factor (*LITAF*) and reduced levels of transforming growth factor-*β4* and *IL-10* compared with the other groups (p<0.05). However, chickens receiving probiotic diets increased *Lactobacillaceae* abundance and reduced *Enterobacteriaceae* abundance in the ceca. Moreover, supplementation with probiotics increased the mRNA expression of *Occludin*, *ZO1*, and *Mucin 2* in the ileum (p<0.05). In addition, probiotic supplementation downregulated the mRNA levels of *IFN-γ* (p<0.05) and *LITAF* (p = 0.075) and upregulated *IL-10* (p = 0.084) expression in the cecal tonsil.

**Conclusion:**

The administration of multi-strain probiotics modulated intestinal microbiota, gene expression of tight junction proteins, and immunomodulatory activity in broiler chickens.

## INTRODUCTION

*Salmonella* species is a key causative agent of salmonellosis in animal products, through which *Salmonella* species enter the food chain [1]. *Salmonella* infection reduces growth performance and causes dysbacteriosis; it even leads to high mortality, which results in huge financial loss in the poultry industry [2]. In the past, antibiotics were widely used to prevent or control *Salmonella* infection in animals. However, the overuse of antibiotic treatments increased drug residues and the emergence of drug-resistant bacteria, which further affected animal health.

Recent studies have focused on evaluating natural sources as potential substitutes for antibiotics, including probiotics and prebiotics, to protect the health status of animals and improve their intestinal microbiota [3]. Probiotics are defined as live, nonpathogenic microbial bacteria that can modulate intestinal microbiota and benefit the host. Probiotic application has been one of the several methods that has reduced *Salmonella* infection in broiler chickens [4,5]. Probiotics can eliminate the colonization of *Salmonella*, enhance intestinal immunity, and strengthen intestinal barrier in the chicken gut [4–6]. In a previous study, we observed that dietary supplementation with multi-strain probiotics improved the intestinal microbiota of chickens and induced different cytokine expression patterns upon *Salmonella* infection [7]. However, few studies have focused on intestinal microbiota, immune parameters, and regulation of mucin and tight junction protein expression in broiler chickens challenged with *Salmonella enterica* subsp. *enterica* and treated with multi-strain probiotics. This study determined the effects of multi-strain probiotics on immune response, gene expression of intestinal tight junction proteins, and cecal microbiota in young broiler chickens using 16S rRNA gene sequencing.

## MATERIALS AND METHODS

### Animals and experimental design

All animal experiments were conducted in compliance with the guidelines of National Chung Hsing University and the Institutional Animal Care and Use Committee (IACUC No. 107-098). All animal manipulations were designed to reduce animal suffering. One-day-old broilers (n = 240) with similar body weights (48.35 g) were divided by gender and randomly allocated to four treatments, each of which had six replicates/pens and 10 birds/pen as follows: *Salmonella* uninfected and non-supplemented group (negative control; Cont), multi-strain probiotic-supplemented group (Pro), *Salmonella*-infected group (Sal), and *Salmonella*-infected and multi-strain probiotic-supplemented group (ProSal). From day 1, chickens in the Cont and Sal groups were fed a basal diet (Table 1), and chickens in the Pro and ProSal groups were fed a probiotic-supplemented basal diet containing multi-strain probiotics, including *Lactobacillus acidophilus* LAP5, *L. fermentum* P2, *Pediococcus acidilactici* LS, and *L. casei* L21 [8]; the concentration of probiotics was 1.0×10^7^ colony forming units (cfu)/g of feed. On day 4, chickens in both the Sal and ProSal groups were gavaged with 1.0×10^8^ cfu/mL of *S. enterica* subsp. *enterica* ST19. Chickens in the Cont and Pro groups received 1.0 mL of sterilized phosphate-buffered saline (pH 7.2) as placebo. Room temperature was controlled at 32°C for the first 3 days and was then gradually reduced to 28°C on the 10th day after hatching. Over the entire experimental period of 10 days, water and feed were provided *ad libitum*.

### Growth performance

Chicks and feed were weighed by cage at the day of hatch and 10 days. Feed consumption was measured, and the body weight gain and feed conversion ratio (FCR) were calculated throughout the entire experimental period.

### DNA sample collection

On day 10, cecal content was collected from an average-size broiler chicken from each pen (six replications/treatment, total n = 24). Samples were stored in an Eppendorf tube at −80°C before bacterial genomic DNA extraction. Total genomic DNA was isolated from 220 mg of frozen cecal content using the QIAamp Fast DNA Stool Mini Kit (Qiagen Inc., Hilden, Germany). DNA concentration and purity were determined using a NanoDrop 2000 spectrophotometer (Thermo Scientific, Waltham, MA, USA).

### Polymerase chain reaction amplification and sequencing

16S rRNA amplicons were measured and pooled for the sequencing reaction. The collection of 16S rRNA sequences was performed using HiSeq 2500, PE250 (Illumina, Inc., San Diego, CA, USA). DNA samples were amplified using the primer set 515F/806R, which targets the V4 region of bacterial 16S rDNA. All polymerase chain reactions were conducted using the Phusion High-Fidelity PCR Master Mix (New England Biolabs, Ipswich, MA, USA). Sequencing libraries were generated using the TruSeq DNA PCR-Free Sample Preparation Kit (Illumina, USA) as per the manufacturer's recommendations, and index codes were added. The library quality was assessed using a Qubit 2.0 Fluorometer (Thermo Scientific, USA) and an Agilent Bioanalyzer 2100 system. Finally, the library was sequenced on an IlluminaHiSeq2500 platform, and 250 bp paired-end reads were generated.

After sequencing, whole tags were assembled using the UCHIME algorithm to detect chimera sequences; the chimera sequences were removed before the effective tags were obtained. Sequence analysis was performed using Uparse software (Uparse v7.0.1001; http://drive5.com/uparse/). Sequences with ≥97% similarity were assigned to the same operational taxonomic units (OTUs). A representative sequence of each OTU was selected for further annotation. Alpha diversity was applied to analyze the complexity of species diversity for a sample by using six indices: Observed outs, Shannon, Simpson, Chao1, abundance-based coverage estimator (ACE), and phylogenetic diversity (PD) whole tree. All the indices of our samples were calculated using QIIME (Version v1.9.1) and were displayed using R software (v3.3.1). To evaluate differences in samples with respect to species complexity, beta diversity analysis on both weighted and unweighted unifrac was conducted using QIIME software (v1.9.1). Partial least squares discriminant analysis (PLS-DA) was also introduced as a supervised model to reveal microbiota variation among groups, which used the “plsda” function in R package “mixOmics” and “ggplot2”. Differential abundance of OTU among treatments was evaluated by metagenomeSeq. The clustered OTUs and taxa information were used for diversity and statistical analyses by Qiime v1.9.1 and R package v.3.3.1 (http://www.R-project.org/). Differences of taxonomic profiles between groups were compared using Statistical Analysis Metagenomic Profiles software v2.1.3 with Welch’s t-test.

### Quantitative polymerase chain reaction

A section of the ileum, jejunum, and cecal tonsil tissue (approximately 20 mg) was aseptically excised and frozen immediately at −80°C until further analyses for gene expression. Tissues were disrupted by homogenization using a homogenizer for 5 min. Total RNA was extracted from the ileum, jejunum, and cecal tonsil samples using Direct-zolTM RNA MiniPrep (Zymo Research, Irvine, CA, USA). The quantity of RNA was measured using the NanoDrop 2000 spectrophotometer (Thermo Scientific, USA). The purity of RNA was verified by measuring absorbance at an optical density ratio of 260 to 280 nm. RNA was normalized to a concentration of 1 μg/μL, after which it was reverse transcribed using a PrimeScript RT Reagent Kit (Takara, Dalian, China) following manufacturer’s instructions. Real-time quantitative polymerase chain reaction (qRT-PCR) was used to quantify the gene expression of the internal standards glyceraldehyde-3-phosphate dehydrogenase (GAPDH) and *β*-actin and cytokines from cDNA samples. PCR reactions were conducted in a total volume of 20 μL containing 10 μL of Power SYBR Green PCR Master Mix (Applied Biosystems, Warrington, UK), 1 μL of cDNA, and 0.25 mM of each primer. The qRT-PCR was performed in duplicate reactions using both forward and reverse primers, cDNA, Power SYBR Green PCR Master Mix (Applied Biosystems, UK), and nuclease-free water. The qRT-PCR was performed using a Step One thermocycler (Applied Biosystem, USA). Pro- and anti-inflammatory cytokines such as interleukin-1β (*IL-1β*), interferon-γ (*IFN-γ*), *IL-6*, lipopolysaccharide-induced tumor necrosis factor-alpha factor (*LITAF*), *IL-10*, and transforming growth factor (*TGF*)-*β4* and tight junction proteins such *Mucin 2*, *Occludin*, *Claudin-1*, and *ZO1* were evaluated for their mRNA expression. The primer pairs used in our study are shown in Table 2. Specific products were amplified using the ABI Step One Real-Time PCR System (Applied Biosystems, UK) with the following temperature–time profiles: 95°C for 30 s, 40 cycles with denaturing at 95°C for 5 s, annealing at 60°C for 20 s, and extension at 72°C for 30 s. Fluorescence was detected at the extension step for each cycle. All the reactions were performed in triplicate. The qRT-PCR data were analyzed using the 2^−^^ΔΔ^^Ct^ method of Livak and Schmittgen [9]. The relative level of each mRNA normalized to the ΔCt (*GAPDH*) and ΔCt (*β-actin*) gene was calculated using the following equation: fold change = 2^Ct target gene (control) – Ct target gene (treatment)^/2^Ct housekeeping gene (control) – Ct housekeeping gene (treatment)^.

### Statistical analysis

Data are presented as mean values with their standard errors and analyzed by two-way analysis of variance to measure the main effects of dietary probiotics and Salmonella challenge using the general linear model procedure of SAS software program (Statistical Analysis System, ver. 8.1; SAS Institute Inc., Cary, NC, USA).

## RESULTS

### Growth and performance

The growth performance results are presented in Table 3. *Salmonella* infection significantly reduced body weight, weight gain, and FCR whereas probiotic addition increased FCR (p<0.05). No significant interactions were found between the *Salmonella* challenge and probiotics on body weight, feed consumption, weight gain, and FCR.

### Microbial diversity

**Figure 1:**
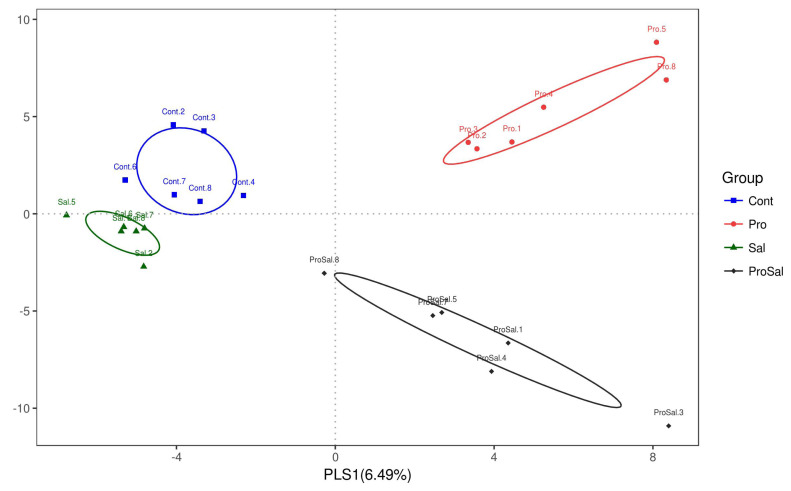
Partial least square discriminant analysis (PLS-DA) loading scatter plot based on the relative abundance of the intestinal microbiota in the cecum of 10-day-old broiler chickens. Cont = uninfected control; Pro = uninfected + 0.1% multi-strain probiotics; Sal = infected *S. enterica* subsp. *enterica*; ProSal. = infected *S. enterica* subsp. *enterica* + 0.1% multi-strain probiotics.

A total of 1,594,639 sequences of the V4 region of the 16S rRNA gene were obtained from the ceca samples. Results of alpha diversity analysis did not show significant main effects of *Salmonella* challenge or probiotic supplementation, but several indices revealed trends of interaction on the microbial community richness and diversity in the ceca of chickens (Table 4). On the other hand, beta diversity analysis (PLS-DA) indicated that bacterial composition in the cecal samples exhibited the tendency of separation in the profiles among the four treatments (Figure 1).

### Diversity and community structure of gut microbiota during chicken development

**Figure 2:**
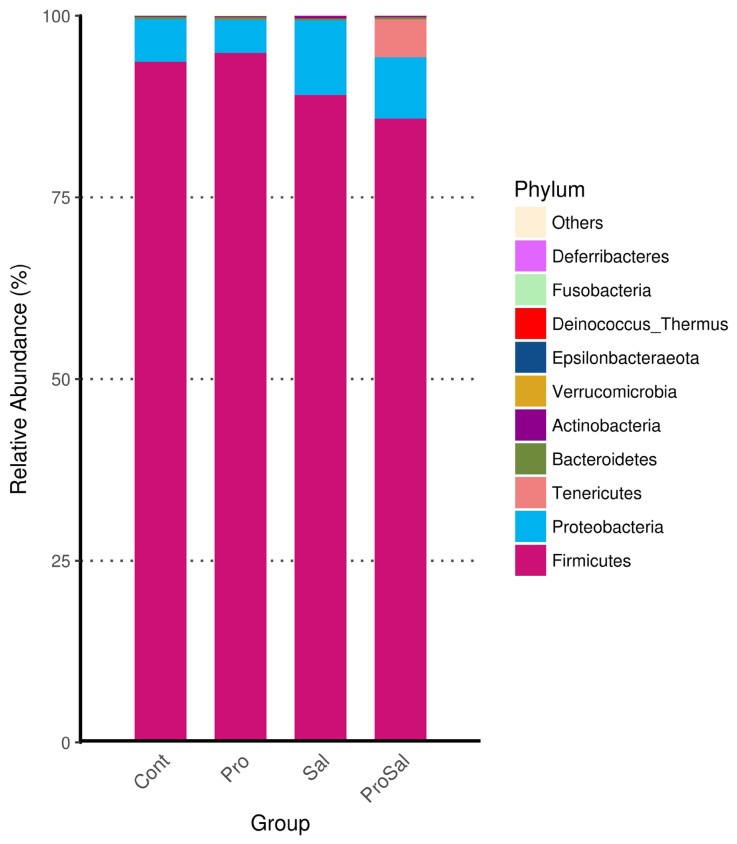
Compositional changes in the cecal microbiota of broiler chickens at the phylum levels. Cont = uninfected control; Pro = uninfected + 0.1% multi-strain probiotics; Sal = infected *S. enterica* subsp. *enterica*; ProSal. = infected *S. enterica* subsp. *enterica* + 0.1% multi-strain probiotics.

**Figure 3:**
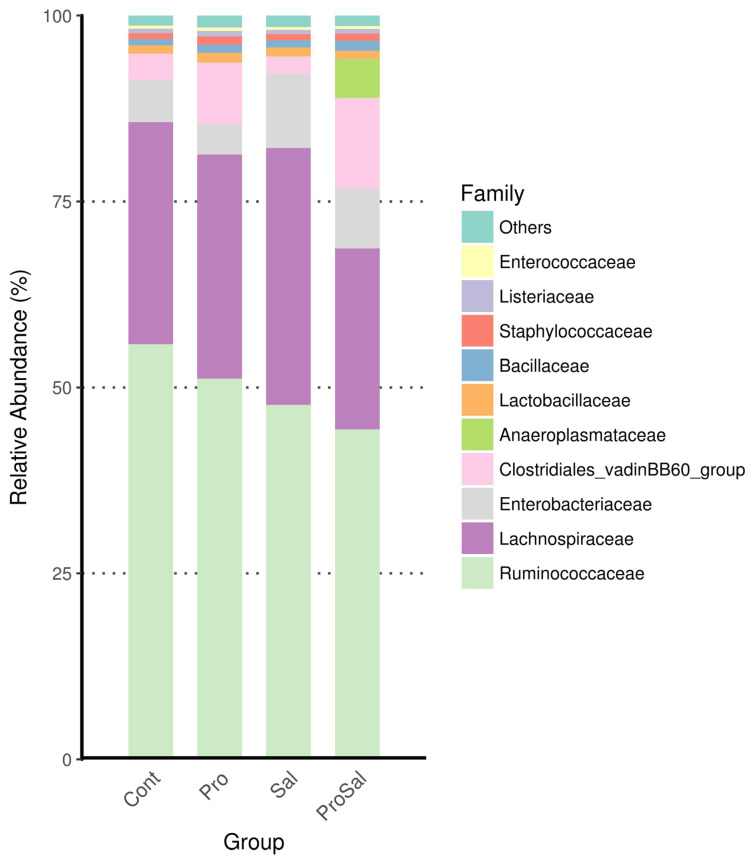
Compositional changes in the cecal microbiota of broiler chickens at the family levels. Cont = uninfected control; Pro = uninfected + 0.1% multi-strain probiotics; Sal = infected *S. enterica* subsp. *enterica*; ProSal. = infected *S. enterica* subsp. *enterica* + 0.1% multi-strain probiotics.

**Figure 4:**
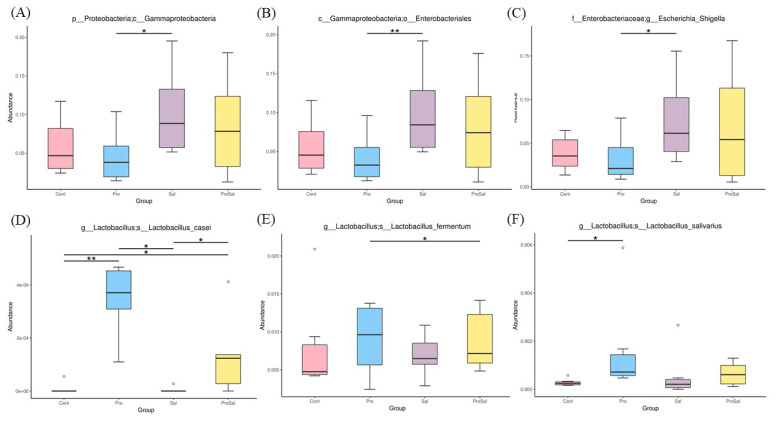
Differential abundance of genera between jejunal and cecal groups analyzed by metagenomeSeq. (A) *Gammaproteobacteria* (B) *Enterobacteriales* (C) *Escherichia-Shigella* (D) *L. casei* (E) *L. fermentum* (F) *L. salivarius* * p≤0.05 and ** p≤0.01. Cont = uninfected control; Pro = uninfected + 0.1% multi-strain probiotics; Sal = infected *S. enterica* subsp. *enterica*; ProSal. = infected *S. enterica* subsp. *enterica* + 0.1% multi-strain probiotics.

Predominant phyla of the cecal microbiota of broiler chickens across all treatments were *Firmicutes* and *Proteobacteria*. The abundance of *Proteobacteria* was lower in the Pro group than in the other groups. The relative abundance of *Proteobacteria* in the Sal group was the highest among the three groups, and it decreased in the ProSal group (Figure 2). In the ceca of broilers, the main bacterial groups were *Ruminococcaceae*, *Lachnospiraceae*, *Enterobacteriaceae Clostridiales* vadinBB60 group, and *Lactobacillaceae* (Figure 3). The abundance of *Lactobacillaceae* in the Pro group was the highest among all treatments. In addition, *Enterobacteriaceae* family decreased in the ProSal group compared to the Sal group (Figure 3). *Salmonella* challenge increased the relative abundance of the taxa of *Gammaproteobacteria*, *Enterobacteriales*, and *Escherichia_Shigella* but decreased the population of *Lactobacillus salivarius* in the ceca. Moreover, the supplementation of probiotics significantly decreased the relative abundance of *Gammaproteobacteria*, E*nterobacteriales*, and *Escherichia shigella*, and increased the amount of *L. casei*, *L. fermentum*, and *L. salivarius* (Figure 4).

### Expression of tight junction protein and cytokine genes

*gene expression, whereas probiotic addition to the diet increased Occludin*, *ZO1* (p<0.001), and *Mucin* 2 (p = 0.019) gene expression, whereas probiotic addition to the diet increased *Occludin* (p = 0.073) gene expression (Table 5). In the ileum, *Salmonella* infection significantly reduced *Occludin* (p<0.001) and *Claudin* (p = 0.022) gene expression, and probiotic supplementation significantly increased the mRNA expression of *Occludin* (p<0.001), *ZO1* (p = 0.005), *Mucin 2* (p<0.001), and *Claudin* (p = 0.051) (Table 6). The gene expression of these selected cytokines in the cecal tonsils was regulated by *Salmonella* infection and probiotic supplementation (Table 7). A significant downregulation of *IFN-γ*, *IL-6*, *IL-1β*, and *LITAF* and upregulation of *TGF-β4* and *IL-10* were observed in the Pro group. *Salmonella* infection significantly increased the expression of *IFN-γ* (p = 0.023), *IL-6* (p<0.001), *IL-1β* (p = 0.002), and *LITAF* (p<0.001) and reduced the expression of *TGF-β4* (p<0.001) and *IL-10* (p = 0.015); however, probiotic supplementation reduced the expression levels of *IFN-γ* (p = 0.045) and *LITAF* (p = 0.075) and increased *IL-10* (p = 0.084) expression. In addition, a significant interaction of *Salmonella* infection across probiotic-supplemented groups was noted for the gene expression of *IFN-γ* (p = 0.037).

## DISCUSSION

Probiotics have been used to replace antibiotics for protecting against and/or reducing pathogen infection in poultry. *Salmonella* is one of the well-known foodborne pathogens that causes poultry infection [10]. The efficacy of probiotics can be evaluated by their effect on growth performance, gut permeability, inflammation, and reduction of pathogenic infection [4,5]. *L. acidophilus* LAP5, *L. fermentum* P2, *P. acidilactici* LS, and *L. casei* L21 were applied in the present study to understand the relationship between multi-strain probiotics and *Salmonella* challenge. Our previous research indicated that the multi-strains probiotics increased relative concentrations of *Firmicutes*, *Lactobacillus*, and *Bifidobacterium* in the intestine [8]. Moreover, the intestinal villi height and short chain fatty acids were increased by supplementation of multi-strains probiotics [8]. The probiotics also regulated immune responses and intestinal microbiota in the specific-pathogen-free chickens against *Salmonella* challenge [7]. Furthermore, several reports have mentioned that *Lactobacillus* spp. prevented *Salmonella*-induced damage to tight junctions and restored intestinal permeability in chickens [5,6]. Thus, apart from intestinal microbial community, we also measured gene expression of tight junction in the present study.

*Salmonella* challenged chickens decreased feed consumption, body weight gain, and FCR, which agreed with several the previous studies [1]. The reduced growth performance observed in the challenged chicken is probably due to the intestinal mucosal damage induced by the *Salmonella* [11]. In contrast, Mountzouris et al [12] reported that *Salmonella*-challenged chickens had similar growth performance as control birds; these contradictory results may be due to discrepancies between the species, strains or dose of *Salmonella* administered, leading to different levels of stabilization of the intestinal environment [13]. In the present study, probiotics supplementation improved broiler chickens FCR. The results were consistent with the findings of other researchers who have observed improvement of growth performance and a reduction of *Salmonella* in the ceca of broilers fed with probiotics [14]. The beneficial effects of probiotic supplements on broiler performance is associated with their role in maintaining healthy balance of bacteria in the digestive tract, intestinal integrity, and improving metabolism [15].

Ecological theory suggests that bacterial species richness is associated with the stability of intestinal microecology. The application of multi-strain probiotics in broiler diets might reduce susceptibility to potential pathogen invasion and reduce intestinal inflammation responses coupled with improvement in intestinal absorption and growth performance of the host [16]. High-throughput sequencing of the V4 region of the 16S rRNA gene was used in the present study to monitor the cecal population of individual broiler chickens. Moreover, PLS-DA is a new tool for the prediction and classification of microarray expression data [17]. Several Alpha-diversity indices, including Observed OTUs, Shannon, Simpson, Chao1, ACE, and PD whole tree, were calculated to reflect the gut microbial community [16]. Although these alpha-diversity indices did not present a significant difference in the cecal microbiota, trends of interaction were observed by observed OTUs (p = 0.114), Chaol (p = 0.078), ACE (p = 0.088), and PD whole tree (p = 0.103). The interaction of alpha diversity might indicate that probiotic could improve microbial community especially on the challenge treatments. Similarly, probiotic significantly changed intestinal microbial community of challenged and non-challenged birds, whereas *Salmonella* infection had little impact on the results of PLS-DA analysis. It should be noticed the birds were challenged with *Salmonella* once (at day 4), while multi-strain probiotics were delivered by feed during whole experiment. Considering the intestinal contents were sampled on 7 days post infection, *Salmonella* challenge might present less influence on intestinal microbial community compared to the probiotic supplementation. Overall, the results suggested that multi-strain probiotic successfully altered intestinal microbiota of birds challenged with *Salmonella*.

The microbiota of broiler chickens has been estimated to surpass 900 bacterial species. The most abundant phylum in the young chicken intestine is *Firmicutes*, followed by *Proteobacteria*, which is consistent with the results of this study (Figure 2) [18]. In the present study, *Salmonella* infection reduced the relative abundance of *Firmicutes* and increased the relative abundance of *Proteobacteria*. Enrichment of the phylum *Firmicutes* and reduction of the phylum *Proteobacteria* were observed after multi-strain probiotic supplementation. The administration of multi-strain probiotics reversed the effects of *Salmonella* infection on phyla *Firmicutes* and *Proteobacteria* richness (Figures 2, 4). The shift in the gut microbial population demonstrated a trend like that reported for *Salmonella* infection in other studies [7]. The phylum *Proteobacteria* includes many pathogens such as *Salmonella*, *Escherichia coli*, and *Shigella*. These pathogens can colonize both humans and chickens and cause intestinal disease. Therefore, increased *Firmicutes* and reduced *Proteobacteria* in the ceca of broilers fed probiotics were associated with improved gut health of broilers in this study. In the present study, chickens in the Sal group had higher abundance of *Lachnospiraceae* and *Enterobacteriaceae* than those in the Cont, Pro, and ProSal groups. Moreover, the Sal group had lower abundance of *Lactobacillaceae* than the Pro group. Many studies have indicated that *Salmonella* infection increased *Enterobacteriaceae* in the ceca of broilers [19]. By contrast, probiotics reduced *Enterobacteriaceae* and increased *Lactobacillaceae*, which produced antimicrobial substances such as hydrogen peroxide, organic acids, and bacteriocins [20].

The intestinal barrier function is important for the animal because it is the first line of protection against pathogen infection. Tight junction proteins are connected to epithelial cells and act as a fence, preventing macromolecular translocation. Our results showed that *Salmonella* infection down-regulated the gene expression of *Occludin* and *Claudin* in the ileum and jejunum of broiler chickens. Similarly, previous studies have indicated that T84 monolayers infected with *S. typhimurium* reduced the expression of *ZO-1* and gut barrier function [5,21]. Similarly, Shao [21] reported that *S. enterica* serovar *typhimurium* infection reduced the expression of *Claudin* and *Occludin* in the jejunum of broiler chickens. Tight junction proteins were correlated with intestinal permeability. The disruption of the intestinal barrier by pathogens allowed the macromolecules such as antigens, bacterial toxins, and pathogens from the intestinal lumen cross into the circulation [22]. The present study indicated that the structures of tight junctions were disrupted by *S. enterica* subsp. *enterica* invasion but were improved by probiotic application. Moreover, the mRNA expression of *Occludin* and *ZO1* in the ileum in probiotic-treated group was higher than that in *Salmonella*-infected group. Wang et al [5] and Wang et al [23] documented that probiotics could improve gut barrier function in IPEC-J2 cells and broiler chickens. Mincun-2 proteins are major components of the chemical barrier, which play an important role in preventing bacterial (enteric) pathogens and various toxins and lubricating the small intestine to maintain mucosal barrier function. Probiotic application can stimulate mucin production, which increases protection against pathogens in the intestine of broilers [24]. However, previous studies have indicated that broilers challenged with *S. typhimurium* exhibited decreased expression of *Mucin 2* [25]. In this study, the gene expression of *Mucin 2* protein was higher in the Pro group than in the Sal group. Our results are consistent with those of other studies that have reported the reinforcement of *Mucin 2* expression following probiotic treatment [24]. Moreover, Liu [26] indicated that the increase in intestinal barrier function by upregulation of tight junction proteins is the key mechanism of probiotic action. The results of the present study indicated that multi-strain probiotic supplementation improved the intestinal epithelial barrier of *Salmonella*-infected broiler chickens through the regulation on gene expression of tight junction proteins. In addition, the intestinal ecosystem is a complex bidirectional interaction system. The improved intestinal microbiota is associated with the integrity of epithelial cells in the gut. The reduced number of cecal *Proteobacteria* and *Enterobacteriaceae* in the ProSal group could be another mechanism through which multi-strain probiotics improved the intestinal barrier of broilers.

Inflammatory cytokines play an important role in the modulation of the intestinal tight junction barrier. Many studies have indicated that the expression of proinflammatory cytokines such as *IFN-γ*, *IL-6*, *IL-1β*, *LITAF*, and anti-inflammatory cytokines (*IL-10* and transforming growth factor-*β*4) is regulated in the cecal tonsils by *Salmonella* infection [7]. Inflammation was increased by *Salmonella* infection (Sal group), and multi-strain probiotics (Pro) reduced inflammation in the cecal tonsils of broiler chickens. *IFN-γ* is a proinflammatory cytokine that was significantly upregulated in chickens after infection with *S. typhimurium* [27, 28]. Adhikari et al [27] indicated that the levels of *IFN-γ* increased in laying hens after infection with 1×10^8^ cfu of *S. typhimurium*. Hsu et al [28] determined that 10-day-old chicks challenged with 10^10^ cfu of *S. typhimurium* showed increased gene expression of *IFN-γ* in cecal tonsils. In this study, the mRNA expression of *IFN-γ* increased in the caecal tonsils of broiler chickens after *S. enterica* subsp. *enterica* infection. Many studies have indicated that probiotic supplementation has anti-inflammatory functions by reducing the level of *IFN-γ* and inflammation and protecting against *Salmonella* in infected chickens [4,7]. We obtained similar results in the present study. The probiotic-associated reduction of *Salmonella* amounts in the intestinal tract reduced *IFN-γ* gene expression in broiler chickens. The *IL-6* is a multifunctional cytokine that acts as both pro-inflammatory and anti-inflammatory cytokine. Cytokine *IL-6* is indicative of the initiation of an acute phase response occurring in avian cells in response to *Salmonella* infection. The challenged 4-day-old specific-pathogen-free chickens with 10^8^ cfu of *S. enterica* subsp. *enterica* and found that 6 days after infection, the expression of *IL-6* mRNA in the cecal tonsils was upregulated when compared with that in the Cont and ProSal group chickens [7]. A similar result was found for our *Salmonella*-infected broiler chickens (Table 5). In this study, we observed that the mRNA expression of *IL-6* in the Cont, Pro, and ProSal groups was decreased compared with that in the Sal group. *LITAF* and *IL-1β* are the key proinflammatory cytokines that regulate host’s immunity against pathogens [29]. Wang et al [5] determined that 1-day-old chicks challenged with 1×10^9^ cfu of S*. typhimurium* had increased gene expression of *LITAF* and *IL-1β* in the cecal tonsils. In this study, the mRNA expression of *LITAF* and *IL-1β* in the ProSal group was numerically lower than that in the Sal group. Whether this is related to changes in the relative abundance of some bacterial taxa in the probiotic-supplemented group remains unclear. However, many studies have reported that different probiotics activate dendritic cells and modulate cytokine production, thus regulating immune responses [8]. *IL-10* and *TGF-β4* have anti-inflammatory properties, and their increased level in pathogen-infected hosts is associated with increased susceptibility to infection [30]. In this study, the mRNA expression of *IL-10* and *TGF-β4* in the Pro group was higher than that in the Sal group. Adhikari et al [27] also reported that probiotics significantly enhanced the expression of *IL-10* and *TGF-β4* genes in *Salmonella*-infected chickens. Accordingly, the increase in *IL-10* and *TGF-β4* expression in the cecal tonsils of chickens challenged with *Salmonella* might be alleviated by adding probiotics in their diets.

## CONCLUSION

The present study suggested that dietary supplementation with a mixture of probiotics reduced the relative abundance of pathogens in the ceca of broiler chickens challenged with *S. enterica* subsp. *enterica*. Moreover, probiotics could protect young broiler chickens from *Salmonella* intestinal disruption by attenuating intestinal inflammation and barrier dysfunction. The interaction of *Salmonella* challenge and probiotics application was not found in most of parameters, indicating that multi-stain probiotic did not only improved gut integrity and intestinal microbial community in the challenged birds, but also in the non-challenge birds. Therefore, dietary supplementation with probiotics is a potentially practical and effective strategy for improving tight junction structure and microbial community of non-challenged birds, as well as controlling the incidence of *Salmonella* infection.

## Figures and Tables

**Table 1:** Ingredients and chemical composition of the experimental diets

Items	Starter diet (1 to 10 days)
Ingredient	g/kg
Corn, yellow	487.8
Soybean meal (CP 44%)	333.0
Full fat soybean meal	100.0
Soybean oil	32.3
Monocalcium phosphate	17.5
Calcium carbonate	16.8
L-lysine-HCl	2.2
DL-methionine	3.7
NaCl	3.9
Choline-Cl	0.8
Vitamin premix1)	1.0
Mineral premix2)	1.0
Total	1,000.0
Calculated nutrient value	
ME (kcal/kg)	3,050
Crude protein (%)	23.02
Calcium (%)	1.05
Total phosphorus (%)	0.74
Available phosphorus (%)	0.50
Lysine (%)	1.43
Methionine+cystein (%)	1.07

ME, metabolizable energy.

1) Supplied per kg of diet: Vit. A 15,000 IU; Vit. D_3_ 3,000 IU; Vit. E 30 mg; Vit. K_3_ 4 mg; riboflavin 8 mg; pyridoxine 5 mg; Vit. B_12_ 25 μg; Ca-pantothenate 19 mg; niacin 50 mg; folic acid 1.5 mg; biotin 60 μg.

2) Supplied per kg of diet: Co (CoCO3) 0.255 mg; Cu (CuSO_4_·5H_2_O) 10.8 mg; Fe (FeSO_4_·H_2_O) 90 mg; Zn (ZnO) 68.4 mg; Mn (MnSO_4_·H_2_O) 90 mg; Se (Na_2_SeO_3_) 0.18 mg.

**Table 2:** Sequence for real-time polymerase chain reaction primers

Gene	Sequence (5′-3′)1)	GenBank Accession
*GAPDH*	F: CCT GCA TCT GCC CAT TT R: GGC ACG CCA TCA CTA TC	NM 204305.1
*β-actin*	F: ACT CTG GTG ATG GTG TTA C R: GGC TGT GAT CTC CTT CTG	NM_205518
*IFN-γ*	F: CTC CCG ATG AAC GAC TTG AG R: CTG AGA CTG GCT CCT TTT CC	Y07922
*IL-6*	F: GCT CGC CGG CTT CGA R: GGT AGG TCT GAA AGG CGA ACA G	AJ250838
*IL-1β*	F: TGG GCA TCA AGG GCT ACA R: TCG GGT TGG TTG GTG ATG	NM_204524
*TGF-β4*	F: AGG ATC TGC AGT GGA AGT GGA T R: CCC CGG GTT GTG TGT TGG T	M31160
*IL-10*	F: CAC AAC TTC TTC ACC TGC GAG R: CAT GGC TTT GTA GAT CCC GTT C	AB559574
*LITAF*	F:TGT GTA TGT GCA GCA ACC CGT AGT R:GGC ATT GCA ATT TGG ACA GAA GT	AY765397
*Mucin 2*	F:TTC ATG ATG CCT GCT CTT GTG R: CCT GAG CCT TGG TAC ATT CTT GT	XM_421035
*Occludin*	F: ACG GCA GCA CCT ACC TCA A R: GGG CGA AGA AGC AGA TGA G	GI:464148
*Claudin-1*	F:CAT ACT CCT GGG TCT GGT TGG T R:GAC AGC CAT CCG ATC TTC T	AY750897.1
*ZO1*	F:CTT CAG GTG TTT CTC TTC CTC CTC R:CTG TGG TTT CAT GGC TGG ATC	XM_413773

*GAPDH*, glyceraldehyde 3-phosphate dehydrogenase; *IFN-γ*, interferon-γ; *IL-1β*, interleukin-1β; *TGF-β4*, transforming growth factor β4; *LITAF*, lipopolysaccharide-induced tumor necrosis factor-alpha factor; *ZO1*, zonula occludens-1.

1) F means forward, R means reverse.

**Table 3:** Effects of dietary probiotic on the growth performance of broiler chickens challenged with *S. enterica* subsp. *enterica*

Items	−*Salmonella c*hallenge1)		+*Salmonella* challenge1)		SEM	p-value		
		
	–Probiotics	+Probiotics	–Probiotics	+Probiotics		*Salmonella* challenge	Probiotics	Salmonella × Probiotics
	
	Cont	Pro	Sal	ProSal.				
d 1–10								
Body weight (g)	241	245	223	234	5.68	0.025	0.236	0.540
Feed consumption (g)	222	216	222	221	4.81	0.663	0.431	0.679
Weight gain (g)	193	197	175	185	5.58	0.023	0.234	0.596
FCR	1.16	1.10	1.27	1.19	0.02	<0.001	0.004	0.529

SEM, standard error of the mean; FCR, feed conversion ratio.

1) Cont = uninfected control; Pro = uninfected + 0.1% multi-strain probiotics; Sal = infected *S. enterica* subsp. *enterica*; ProSal. = infected *S. enterica* subsp. *enterica* + 0.1% multi-strain probiotics.

**Table 4:** Diversity indices of the caecal microbiota of broiler chickens

Group	−*Salmonella* challenge		+*Salmonella* challenge		SEM	p-value		
		
	–Probiotics	+Probiotics	–Probiotics	+Probiotics		*Salmonella* challenge	Probiotics	*Salmonella* × Probiotics
	
	Cont1)	Pro1)	Sal1)	ProSal.1)				
Diversity indices								
Observed otus	196	211	199	216	4.88	0.724	0.933	0.114
Shannon	4.98	5.03	4.85	5.02	0.07	0.719	0.681	0.471
Simpson	0.94	0.94	0.94	0.94	0.00	0.991	0.452	0.955
Chao1	229	241	234	244	2.57	0.386	0.460	0.078
ACE	232	237	232	245	3.00	0.915	0.472	0.088
PD whole tree	4.64	4.86	4.63	4.92	0.07	0.824	0.850	0.103

SEM, standard error of the mean; ACE, abundance-based coverage estimator; PD, phylogenetic diversity.

1) Cont = uninfected control; Pro = uninfected + 0.1% multi-strain probiotics; Sal infected *S. enterica* subsp. *enterica*; ProSal. = infected *S. enterica* subsp. *enterica* + 0.1% multi-strain probiotics.

**Table 5:** Effects of dietary probiotic on the relative expressions of tight junction protein in the jejunum of broiler chickens challenged with *S. enterica* subsp. *enterica*

Items	−*Salmonella* challenge		+*Salmonella* challenge		SEM	p-value		
		
	–Probiotics	+Probiotics	–Probiotics	+Probiotics		*Salmonella* challenge	Probiotics	*Salmonella* × Probiotics
	
	Cont1)	Pro1)	Sal1)	ProSal.1)				
*Occludin*	1.03	1.21	0.73	0.92	0.08	<0.001	0.073	0.939
*ZO1*	1.02	1.05	0.93	1.01	0.08	0.479	0.514	0.771
*Mucin 2*	1.05	0.88	0.53	0.80	0.10	0.019	0.662	0.079
*Claudin*	1.08	1.14	0.88	0.95	0.03	0.142	0.624	0.974

SEM, standard error of the mean; *ZO1*, zonula occludens-1; *GAPDH*, glyceraldehyde 3-phosphate dehydrogenase.

1) Cont = uninfected control; Pro = uninfected + 0.1% multi-strain probiotics; Sal = infected *S. enterica* subsp. *enterica*; ProSal. = infected *S. enterica* subsp. *enterica* + 0.1% multi-strain probiotics.

*GAPDH* and *β-actin* were used as housekeeping genes, and the 2^−^^ΔΔ^^Ct^ method was used to determine the relative abundance.

**Table 6:** Effects of dietary probiotic on the relative expressions of tight junction protein in the ileum of broiler chickens challenged with *S. enterica* subsp. *enterica*

Items	−*Salmonella* challenge		+*Salmonella* challenge		SEM	p-value		
		
	–Probiotics	+Probiotics	–Probiotics	+Probiotics		*Salmonella* challenge	Probiotics	*Salmonella* × Probiotics
	
	Cont	Pro	Sal	ProSal.				
*Occludin*	1.01	1.41	0.41	0.86	0.08	<0.001	<0.001	0.730
*ZO1*	1.01	1.17	0.94	1.22	0.06	0.856	0.005	0.434
*Mucin 2*	1.01	1.41	0.94	1.23	0.07	0.146	<0.001	0.527
*Claudin*	1.02	1.46	0.85	0.97	0.12	0.022	0.051	0.257

SEM, standard error of the mean; *ZO1*, zonula occludens-1; *GAPDH*, glyceraldehyde 3-phosphate dehydrogenase.

1) Cont = uninfected control; Pro = uninfected + 0.1% multi-strain probiotics; Sal = infected *S. enterica* subsp. *enterica*; ProSal. = infected *S. enterica* subsp. *enterica* + 0.1% multi-strain probiotics.

*GAPDH* and *β-actin* were used as housekeeping genes, and the 2^−^^ΔΔ^^Ct^ method was used to determine the relative abundance.

**Table 7:** Effects of dietary probiotic on the expressions of inflammation-related genes in the cecal tonsils of broiler chickens challenged with *S. enterica* subsp. *enterica*

Items	−*Salmonella* challenge		+*Salmonella* challenge		SEM	p-value		
		
	–Probiotics	+Probiotics	–Probiotics	+Probiotics		*Salmonella* challenge	Probiotics	*Salmonella* × Probiotics
	
	Cont1)	Pro1)	Sal1)	ProSal. 1)				
*IFN-γ*	1.05	1.07	1.93	1.11	0.17	0.023	0.045	0.037
*IL-6*	1.07	1.13	2.46	1.79	0.18	<0.001	0.168	0.102
*IL-1β*	1.06	0.94	2.75	1.98	0.34	0.002	0.288	0.431
*LITAF*	1.07	0.88	1.80	1.39	0.15	<0.001	0.075	0.497
*TGF-β4*	1.08	1.36	0.65	0.66	0.14	<0.001	0.359	0.352
*IL-10*	1.07	1.35	0.85	0.98	0.10	0.015	0.084	0.508

SEM, standard error of the mean; *IFN-γ*, interferon-γ; *IL*, interleukin; *LITAF*, lipopolysaccharide-induced tumor necrosis factor-alpha factor; *TGF-β*4, transforming growth factor β4; *GAPDH*, glyceraldehyde 3-phosphate dehydrogenase.

1) Cont = uninfected control; Pro = uninfected + 0.1% multi-strain probiotics; Sal = infected *S. enterica* subsp. *enterica*; ProSal. = infected *S. enterica* subsp. enterica + 0.1% multi-strain probiotics.

*GAPDH* and *β-actin* were used as housekeeping genes, and the 2^−^^ΔΔ^^Ct^ method was used to determine the relative abundance.
